# Intolerance of Uncertainty and Anxiety (but not Alexithymia) Mediate the Association Between Autistic Traits and Quality of Life

**DOI:** 10.1007/s10803-024-06310-9

**Published:** 2024-03-06

**Authors:** Yeju Lin, David Mason, Colette Hirsch, Francesca Happé

**Affiliations:** 1 Social, Genetic & Developmental Psychiatry Centre, Institute of Psychiatry, Psychology and Neuroscience, King’s College, London, UK; 2 Department of Psychology, Institute of Psychiatry, Psychology and Neuroscience, King’s College, London, UK

**Keywords:** Autism, Autistic traits, Alexithymia, Intolerance of uncertainty, Anxiety, Quality of life, Mediation

## Abstract

Previous research has indicated that autistic individuals report lower quality of life (QoL) than non-autistic people. It is unclear whether it is the autism traits themselves or co-occurring thinking styles or mental health difficulties that most impair QoL. This study tested a hypothesised model to explore how ‘intolerance of uncertainty’ (IU), alexithymia (difficulty in identifying and describing own emotions), and anxiety play into the association between autistic traits and QoL. Online survey data were analysed from 116 autistic and 51 non-autistic adults who completed six standardised questionnaires measuring autistic traits, alexithymia, IU, anxiety and QoL (physical health, psychological health, social relations, and environment domains). The autistic group reported higher scores for alexithymia, IU and anxiety, and lower scores for QoL across domains, compared to the non-autistic group. Across the entire sample, autistic traits, alexithymia, IU and anxiety were positively correlated with one another, and negatively related to the four domains of QoL. Finally, IU and anxiety partially serially mediated the pathways from autistic traits to physical health and environment domains of QoL, and fully mediated the pathways from autistic traits to psychological health and social relations domains of QoL, across the full sample. The lower QoL experienced by autistic people may be explained in part by the mediating effect of both IU and anxiety (but not alexithymia). This study highlights the need for evidence-based interventions to address both IU and anxiety to improve QoL for autistic people/those with high levels of autistic traits.

## Introduction

Autism Spectrum Disorder (ASD) is a lifelong neurodevelopmental condition characterised and diagnosed by atypicalities in social communication and interaction, and restricted and repetitive behaviours and interests (American Psychological Association, 2022). The prevalence of autism is estimated at 1% in the general population in the UK (Baird et al., 2006; Brugha et al., 2016). It has been found that, from a systematic review of 14 studies, adults on the autism spectrum reported significantly lower quality of life (QoL) relative to neurotypical (NT) individuals, using different assessment tools for QoL (Ayres et al., 2018). QoL is a multi-dimensional concept (Theofilou, 2013) describing “individuals’ perception of their position in life in the context of the culture and value systems in which they live and in relation to their goals, expectations, standards and concerns” (WHOQOL Group, 1995, p.1403). Beyond clinical diagnosis, autistic traits have been found to correlate negatively with QoL across the four domains of physical health, psychological health, social relations and environment (e.g., Capp et al., 2022). The exploration of how higher autistic traits link to lower QoL is important, as poorer QoL persists across the lifespan in autistic individuals (Van Heijst & Geurts, 2015).

Relative to the non-autistic peers, autistic children and adults have elevated rates of co-occurring mental health difficulties (Lai et al., 2019), which have been found to decrease autistic people’s QoL. In particular, anxiety symptoms significantly and negatively impact autistic people’s daily life (South et al., 2017). The current and lifetime rate of all anxiety disorders in autistic adults is estimated at 27% and 42%, respectively (Hollocks et al., 2019). Autistic symptoms, including social skills and sensory sensitivities, are significantly related to anxiety in autistic people across the age range (Boulter et al., 2014; Hwang et al., 2020; Maisel et al., 2016; South et al., 2021; Uljarević et al., 2016). Compared to NT individuals, autistic people report higher scores on trait anxiety (Jolliffe et al., 2022), and these are negatively associated with self-reported quality of life (QoL) (Sáez-Suanes & Álvarez-Couto, 2022). Older autistic adults with self-reported anxiety also report significantly poorer QoL than autistic peers without anxiety (Mason et al., 2019). A recent study found that higher self-report anxiety correlated significantly with lower global QoL scores in autistic and non-autistic adults. The same study reported that, compared to older autistic adults (aged 50–71 years), younger autistic adults (aged 19–48 years) were more likely to meet the clinical cut-off for self-reported anxiety symptoms, and had significantly lower scores on the social relations domain of QoL (Yarar et al., 2022). So, anxiety is strongly associated with autistic people’s QoL.

Other factors influencing autistic people’s QoL may be aspects of thinking style which are common in autism but not part of the diagnostic criteria or core features. An example, itself strongly linked to anxiety more generally, is intolerance of uncertainty (IU). IU has been characterised as an excessive tendency to believe that changes, surprises and uncertainty about the future are threatening and unacceptable without considering their probabilities (Carleton, 2012). The cognitive bias will trigger negative cognitive, emotional and behavioural reactions to uncertain situations and events in daily life (Freeston et al., 1994). A meta-analysis of 10 studies found that IU was significantly associated with anxiety (with large effect size) in autistic individuals aged 4–70 years (Jenkinson et al., 2020). After controlling for anxiety symptoms, there was still a significant association between IU and autism symptoms (difficulties in social communication, and repetitive behaviours; Vasa et al., 2018). Additionally, IU has been found to be a significant predictor of QoL in autistic children and their carers (Adams et al., 2019; Sonido et al., 2022). In a qualitative study, 53 caregivers of autistic children reported that IU had an impact on not only their children’s but also their families’ QoL (Goodwin et al., 2022). Hence, IU is associated with anxiety, autistic features and QoL.

Alexithymia, a difficulty in identifying and describing one’s own emotions (Sifneos, 1973), is also an important putative factor for autistic people’s mental health and QoL. A meta-analysis of 15 published studies concluded that autistic individuals have a significantly higher prevalence rate (49.9%) of alexithymia than non-autistic comparison groups (Kinnaird et al., 2019). Furthermore, alexithymia has been found to be a significant mediator impacting the relationship between autistic traits and anxiety symptoms in autistic and non-autistic adults (Barros et al., 2022; Maisel et al., 2016). In particular, the autism features of social impairment and restricted interests and detail orientation demonstrated an indirect effect on anxiety, mediated through the alexithymia domain of ‘difficulty in identifying emotion’ (Barros et al., 2022). In addition, IU has been found to mediate the pathway from alexithymia to internalising problems (e.g., anxiety) in a sample of 95 autistic children (Ozsivadjian et al., 2021). A negative correlation between alexithymia and physical health and psychological health domains of QoL has been reported in a sample of 42 autistic participants and 91 non-autistic participants in the UK (Mason & Happé, 2022). Therefore, anxiety, IU and alexithymia are three important co-occurring mental health factors/thinking styles that may underlie the association between autistic traits and QoL. Indeed, there is good reason to think that factors associated with autism, rather than autism per se, are responsible for poor QoL in autistic groups (Happè & Frith, 2020). For example, not all autistic individuals report low QoL (Oakley et al., 2021), and autism symptom severity does not predict autistic people’s QoL across the lifespan (Van Heijst & Geurts, 2015).

**Fig. 1:**
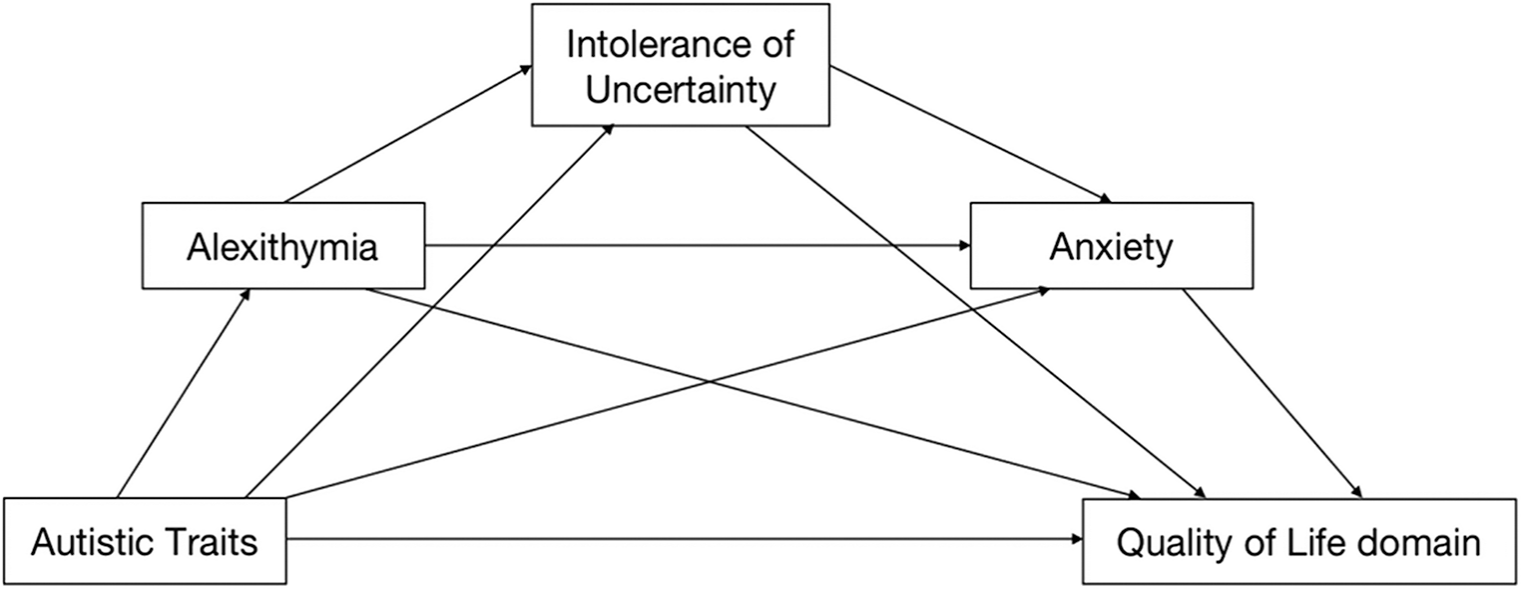
Hypothesised model of association between autistic traits and quality of life domains via alexithymia, intolerance of uncertainty and anxiety

Hence, the current study aimed to examine the associations between autistic traits, anxiety, IU, alexithymia and QoL. Based on previous evidence from mediation analysis, IU and alexithymia are significant mediators of the relationship between autistic traits and anxiety symptoms in autistic and non-autistic individuals (Boulter et al., 2014; Hwang et al., 2020; Maisel et al., 2016; Neil et al., 2016; Sáez-Suanes et al., 2020; South et al., 2021). IU has also been shown to mediate the association between alexithymia and anxiety in autistic children and adults (Moore et al., 2022; Ozsivadjian et al., 2021). Therefore, the present study tested a hypothesised model, shown in Fig. 1, using a sample of autistic and non-autistic adults. We predicted that (1) the autistic sample will report significantly higher scores for alexithymia, IU and anxiety, and lower scores for the four domains of QoL in comparison with the non-autistic sample; (2) there will be significant positive correlations between autistic traits, alexithymia, IU, and anxiety, while all these variables will be negative associated with the four domains of QoL in the total (i.e., autistic plus non-autistic) sample; (3) the association between autistic traits and QoL will be mediated by alexithymia, IU and anxiety in the total sample.

## Methods

### Participants

The present study used data previously collected for a study (unpublished) about autistic adults’ experiences of moving home in the UK. Recruiting through the Autistica Network and Twitter, potential participants were included in the original study if they were over the age of 18, living in the UK, with or without a diagnosis of autism, and have access to internet and the ability to complete an online survey. A total of 266 participants contributed data in response to the original online survey via Qualtrics (2020). Of these, 167 participants completed all the questionnaires analysed in the present study. The completion rate was therefore 62.8%, and those who completed versus skipped the questionnaires were on average older (58.3% aged above 30 years, Mann-Whitney U test *p* = .004), and more likely to report a formal diagnosis of autism (*p* < .001). Participants completing the entire survey and participants skipping questionnaires did not differ in gender distribution.

The final sample of 167 participants consisted of 40 male (24.0%), 107 female (64.1%), 13 non-binary/gender fluid (7.8%) and 7 other responses (e.g., “don’t know”, “don’t care”, “agender/neutrois”), including no response (4.2%). The mean age was 37.6 years (*SD* = 13.9), with ages ranging from 18 to 76 years. More than half (*N* = 102, 61.1%) of the participants reported having a formal diagnosis of autism, 14 participants (8.4%) self-identified as autistic but without a formal diagnosis, and 51 participants (30.5%) neither had a formal autism diagnosis nor self-identified as autistic. All the participants completed two questionnaires regarding autistic traits: the 10-item Autism Spectrum Quotient (AQ-10; Booth et al., 2013) and the 14-item Ritvo Autism & Asperger Diagnostic Scale (RAADS-14; Eriksson et al., 2013). In the present sample, the group reporting a formal autism diagnosis and those who self-identified as autistic did not differ in mean scores on AQ-10 (*M(SD)*_(autistic)_ = 8.18(1.90), *M(SD)*_(self−identified)_ = 8.29(1.54); ${\eta }^{2}$ = 0.58) and RAADS-14 (*M(SD)*_(autistic)_ = 34.41(7.82), *M(SD)*_(self−identified)_ = 33.71(5.05); ${\eta }^{2}$ = 0.58). Therefore, the 14 self-identified autistic participants were added into the autistic group. The demographic characteristics of the autistic and non-autistic groups are shown in Table 1. The autistic group was slightly older and had significantly more individuals that reported high autistic traits (measured by AQ-10 and RAADS-14) than the non-autistic group. However, the two groups had similar ratios for gender, intellectual disability and employment status (though, there was missing data for some participants).

**Table 1:** Demographic characteristics of autistic and non-autistic samples

	autistic (*N* = 116)	non-autistic (*N* = 51)	p	effect size
Age in years, M (SD)	38.97 (12.58)	34.35 (16.24)	0.002^*^	.24^a^
Gender			0.169	.17^b^
Male, N (%)	27 (25.5%)	13 (23.3%)		
Female, N (%)	71 (61.2%)	36 (70.6%)		
Non-binary/ Gender fluid, N (%)	11 (9.5%)	2 (3.9%)		
Other/missing, N (%)	7 (6.0%)	0 (0.0%)		
AQ-10 autistic traits			< 0.001^*^	.83^b^
High autistic traits N (%)	105 (90.5%)	2 (3.9%)		
Low autistic traits N (%)	11 (9.5%)	49 (96.1%)		
RAADS-14 autistic traits			< 0.001^*^	.87^b^
High autistic traits N (%)	114 (98.3%)	7 (13.7%)		
Low autistic traits N (%)	2 (1.7%)	44 (86.3%)		
Intellectual disability			0.221	.09^b^
With intellectual disability N (%)	4 (3.4%)	4 (7.8%)		
Without intellectual disability N (%)	112 (96.6%)	47 (92.2%)		
Other mental health conditions			0.632	.05^b^
Did not report	75 (64.7%)	29 (56.9%)		
One conditions	17 (14.7%)	9 (17.6%)		
More than one conditions	24 (20.7%)	13 (25.5%)		
Employment status (*N* = 122)			0.338	.09^b^
Employed	61 (64.2%)	20 (74.1%)		
Unemployed	34 (35.8%)	7 (25.9%)		

*Note* “*p*” = significance value for group comparison between autistic and non-autistic samples.

^a^ Effect size r for Mann-Whitney U test: < 0.3 = small, 0.3–0.5 = medium, > 0.5 = large (Karadimitriou et al., 2018).

^b^ Effect size Cramer’s V for chi-square test with one degree of freedom: 0.1–0.3 = small, 0.3–0.5 = medium, larger than 0.5 = large (Zach, 2020).

^*^p < .05

### Measures

#### 10-Item Autism Spectrum Quotient (AQ-10: Booth et al., 2013)

The AQ-10 is an abbreviated 10-item version of the original 50-item Autism Spectrum Quotient assessing present autistic traits in adults (Booth et al., 2013). Example items are “I often notice small sounds when others do not”, and “I find it easy to do more than one thing at once”. The response scale ranges from *1 (definitely disagree)* to *4 (definitely agree)*. For items 1, 7, 8 and 10, agreeing (definitely or slightly) scores one point; while for items 2–6 and item 9, disagreeing (definitely or slightly) scores one point. The overall score ranges from 0 to 10, and a cut-off of six has shown good sensitivity and specificity for autistic adults (Booth et al., 2013). In the present study, Cronbach’s alpha was 0.87 for the total sample, 0.66 for the autistic subsample and 0.53 for the non-autistic subsample. Due to the poor reliability, the total score on the AQ-10 was used solely to characterise the groups in the current study.

#### 14-Item Ritvo Autism & Asperger Diagnostic Scale (RAADS-14; Eriksson et al., 2013)

The RAADS-14 is a short version of the 80-item Ritvo Autism and Asperger Diagnostic Scale-Revised (RAADS-R), which measures autistic traits both in the present and the past (Eriksson et al., 2013). It consists of 14 items in three domains: Mentalizing Deficits, Social Anxiety and Sensory Reactivity. Example items for the three domains, respectively, include: “I focus on details rather than the overall idea”, “How to make friends and socialize is a mystery to me”, and “When I feel overwhelmed by my senses, I have to isolate myself to shut them down”. Responses are made on a four-point Likert scale, i.e., *0 (‘never true’)*, *1(‘true only when I was younger than 16’)*, *2 (‘true only now’)* and *3 (‘true now and when I was young’)*. One item (6) is reverse scored. The scores of all items are summed to a total score, with a cut-off of 14 considered to indicate high autistic traits (Eriksson et al., 2013). The RAADS-14 has shown acceptable internal consistency and construct validity in clinical and non-clinical samples (Eriksson et al., 2013). In the present study, Cronbach’s alpha was 0.95 for the total sample and 0.82 for both the autistic and non-autistic subsamples. Because of the better internal consistency, compared to the AQ-10 (in our data and previous reports; Bertrams & Shah, 2021; Taylor et al., 2020), the total score on the RAADS-14 was used to capture autistic traits in the correlations and mediation models reported in the Results below.

#### 20-Item Toronto Alexithymia Scale (TAS-20; Bagby et al., 1994a, b)

TAS-20, a revised version of the original TAS, is a 20-item self-report measure of alexithymia (Bagby et al., 1994a, b). There are three subscales: Difficulty Describing Feelings, Difficulty Identifying Feeling, and Externally Oriented Thinking. Example items are “I am often confused about what emotion I am feeling”, “I find it hard to describe how I feel about people”, and “I prefer to analyze problems rather than just describe them”. Responses are made on a five-point Likert scale with scores from *1 (strongly disagree)* to *5 (strongly agree)*. Items 4, 5, 10, 18 and 19 are reverse scored. The total scores were used in the present study, with higher scores indicating more traits of alexithymia. Good psychometric properties have been reported for the TAS-20 in general population and behavioural medicine population (Bagby et al., 1994a, b, 2020). However, Williams and Gotham (2021) reported that the structural validity of the TAS-20 was inadequate in a large sample of autistic and non-autistic individuals, and suggested instead a revised 8-item version of the scale (Generalised Alexithymia Factor Score; GAFS-8) with good psychometric properties. In the present study, Cronbach’s alpha for the TAS-20 were 0.91, 0.85 and 0.87, and for the GAFS-8 were 0.91, 0.84 and 0.85 in the total sample, the autistic subsample and the non-autistic subsample, respectively. Due to the similar internal consistency in the present study and wider use of the TAS-20 in previous studies, the TAS-20 was used as the main measure of alexithymia, although results using the GAFS-8 are reported as supplementary analyses for the interested reader.

#### 12-Item Intolerance of Uncertainty Scale (IUS-12; Carleton et al., 2006)

The IUS-12 is a shorter version of the original 27-item IUS measuring the construct of IU (Carleton et al., 2006). It has two subscales; Prospective IU and Inhibitory IU. Example items are “Unforeseen events upset me greatly”, and “Uncertainty keeps me from living a full life”. The response scale ranges from *‘not at all characteristic of me (1)’* to *‘entirely characteristic of me (5)’*. Item scores are summed to a total score, with higher scores indicating greater IU. The IUS-12 has shown sufficient structural validity, internal consistency, test-retest reliability, convergent validity and known-groups validity in clinical anxiety samples and non-clinical samples (Carleton et al., 2006; Wilson et al., 2020). In the present study, Cronbach’s alpha for the IUS-12 was 0.95 for the total sample, 0.91 for the autistic subsample and 0.89 for the non-autistic subsample.

#### 7-Item Generalised Anxiety Disorder Assessment (GAD-7; Spitzer et al., 2006)

The GAD-7 is a brief clinical assessment tool for symptoms of generalised anxiety disorder (GAD), assessed over the last two weeks (Spitzer et al., 2006). The seven items are responded to on a four-point Likert scale, from *0 (not at all)* to *3 (nearly every day)*. Example items are “Feeling nervous, anxious or on edge”, and “Trouble relaxing”. Item scores are summed with a max score of 21, and a suggested cut-off for probable GAD is 10 (Spitzer et al., 2006). Previous research supports the good internal consistency, test-retest reliability and construct validity of the GAD-7 (Spitzer et al., 2006). In the present study, Cronbach’s alpha for the GAD-7 was 0.89 for the total sample, 0.88 for the autistic subsample and 0.83 for the non-autistic subsample.

#### 26-Item World Health Organization Quality-of-Life Scale (WHOQOL-BREF; WHOQOL Group, 1998)

The WHOQOL-BREF, assessing individuals’ quality of life, is a 26-item version of the original full version World Health Organization Quality-of-Life Scale (WHOQOL-100; WHOQOL Group, 1998). It contains two global questions and four subscales (domains; example questions shown): Physical Health (“Do you have enough energy for everyday life?”), Psychological Health (“How much do you enjoy life?”), Social Relations (“How satisfied are you with your personal relationships?”), and Environment (“How safe do you feel in your daily life?”). Respondents answer on a five-point Likert scale from *1 (very poor/very dissatisfied/not at all/never)* to *5 (very good/very satisfied/extremely/always)*. Items 3, 4 and 26 are reverse scored. There are four final scores, transforming from the raw scores to a 0-100 scale, for the four domains captured by the subscales (World Health Organization, 1996). The WHOQOL-BREF has been reported to have acceptable structural validity, internal consistency, test-retest reliability, convergent and discriminant validity in large datasets (WHOQOL Group, 1998), and good psychometric properties have also been reported for autistic people (McConachie et al., 2018). In the present study, Cronbach’s alpha for the four domains ranged from 0.70 to 0.87 for the total sample and the autistic and non-autistic subsamples. Each of the domain scores was used in mediation models below.

### Statistical Analysis

All data analyses were conducted using SPSS software (version 28.0; IBM Corp., 2021). The maximum missing data proportion was 3.0% for the item 21 of WHOQOL-BREF, and the lowest missing data proportion was 0.6% for the item 2 of GAD-7. Checking Little’s test (Little, 1988) and the missing data pattern, missing data were missing at random but not completely at random. All the missing data points were replaced by the expectation-maximisation algorithm (Moon, 1996). To test the internal consistency of each instrument, Cronbach’s alpha (Tavakol & Dennick, 2011) was used in the total sample as well as in the autistic/non-autistic subsamples, as reported above; an alpha of 0.70 or above is considered acceptable.

For descriptive analysis, frequencies and percentages were calculated for the categorical demographic variables; means, standard deviation (*SD*), range, skewness and kurtosis were calculated for continuous demographic variables and outcome variables. As the sample size was more than 50, Kolmogorov-Smirnov (*K-S*) test with Lilliefors significance correction were conducted to examine the assumption of normality for each continuous variable (Ghasemi & Zahediasl, 2012). Due to the non-normal distribution of the data, non-parametric tests, i.e., Mann-Whitney U-Test (Nachar, 2008), Kruskal-Wallis H-Test (Chan & Walmsley, 1997) and Spearman’s rank correlation (Zar, 2005), were used to investigate group differences and correlations. For post hoc comparison, significance value was adjusted by Bonferroni correction (Ranstam, 2016). To test the difference between categorical variables, Pearson chi-square test (Sharpe, 2015) was conducted. Effect size for group comparison was calculated by *r* (Karadimitriou et al., 2018), eta-square (${\eta }^{2}$; Tomczak & Tomczak, 2014) and Cramer’s V (Vrbin, 2022) for Mann-Whitney U-Test, Kruskal-Wallis H-Test and Pearson chi-square test, respectively.

For mediation analysis, our hypothesised model was examined by Hayes’ model six - a serial model with three mediators - using Hayes’ version four of PROCESS macro (Hayes, 2017). Hayes’ PROCESS is the non-parametric bootstrapping method, which does not require that the variables follow a specific distribution for the purpose of estimating the confidence intervals. As the dependent variable - quality of life - had four domains, four serial models with three mediators were conducted. In each model, there were ten direct effects, seven indirect effects and one total effect. The confidence intervals were set at 95%, and the number of bootstrap samples was 5000. The linearity, multicollinearity and homoscedasticity of the data were screened by scatter plot, tolerance and variance inflation factor (*VIF*), and residual (scatter) plot, respectively. The post hoc power analysis for the sample size was tested by Monte Carlo Power Analysis for mediation models (Schoemann et al., 2017). The final effect size of each indirect effect was examined by *P*_*M*_ - the fraction of the indirect effect divided by the total effect (Wen & Fan, 2015).

### Ethics

The present study used an existing dataset which was collected online via the survey platform Qualtrics (2020). Original data collection received ethical approval (reference number HR-19/20-17363), and the processes and analyses presented here were further approved by the Health Faculties Research Ethics Sub-Committee of the Institute of Psychiatry, Psychology & Neuroscience at the University of King’s College London (reference number LRS/DP-21/22-30313). All data were processed in accordance with the General Data Protection Regulation 2016.

## Results

### Between Group Differences

Supporting the first hypothesis, the autistic group presented significantly higher scores for alexithymia, IU and anxiety, and lower scores for physical health, psychological health, social relations and environment domains of quality of life, relative to the non-autistic group. Table 2 displays the means, standard deviations and the minimum and maximum values of the scores in the autistic and non-autistic subgroups, as well as the significance of group differences from the Mann-Whitney U-Test.

### Correlations Between Variables

Spearman’s correlation coefficients (and Cronbach’s alpha) for the key variables are displayed in Table 3. Supporting the second hypothesis, autistic traits, alexithymia, IU and anxiety were strongly and positively correlated with each other (*r* = .44 to 0.78, *p* < .001), and negatively related to all four quality of life domains (*r* = -.33 to -0.70, *p* < .001). Therefore, all the key variables were entered into the mediation analysis testing the hypothesised model. Age was significantly positively associated with autistic traits as assessed by the RAADS-14 (but not the AQ-10), and was significantly negatively correlated with two domains of QoL (physical health and social relations). Age was therefore included as a covariate in the subsequent mediation models for physical health and social relations QoL. Gender had a significant effect on physical health (*H* = 8.37, *p* = .04) and alexithymia (*H* = 8.42, *p* = .04), but after Bonferroni correction, the only significant differences were lower physical health QoL in non-binary/gender fluid group compared to females (*t* = 39.06, *adj. p* = .04), and higher alexithymia scores in the non-binary/gender fluid group compared to the male (*t* = -43.24, *adj. p* = .03) and female groups (*t* = -38.73, *adj. p* = .04). Given the very small number (*n* = 13) of participants in the non-binary/gender fluid group, gender was not included as a covariate in the subsequent analyses.

**Table 2:** Scores for alexithymia, intolerance of uncertainty, anxiety and the four domains of quality of life, by group; means (SD) and the minimum and maximum values

	autistic (*N* = 116)	non-autistic (*N* = 51)	Z	effect size^a^
Alexithymia (max = 100)	62.43 (11.81) *33–86*	41.22 (11.07) *21–71*	6.84^***^	0.53
Intolerance of Uncertainty (max = 60)	45.00 (10.01) *22–60*	25.86 (7.98) *13–48*	8.63^***^	0.67
Anxiety (max = 21)	12.44 (5.61) *1–21*	6.47 (4.48) *0–20*	6.08^***^	0.47
Quality of Life				
Physical Health (max = 100)	54.14 (21.04) *4-100*	78.50 (14.52) *39–100*	-6.70^***^	0.52
Psychological Health (max = 100)	41.45 (21.25) *0–88*	61.60 (16.10) *25–96*	-5.50^***^	0.43
Social Relations (max = 100)	48.33 (24.63) *0-100*	68.23 (21.08) *17–100*	-4.80^***^	0.37
Environment (max = 100)	57.25 (21.03) *6-100*	79.17 (12.03) *53–100*	-6.50^***^	0.50

*Note* Numbers in parentheses are standardised deviations; numbers in italic format are the minimum and maximum values.

^a^ Effect size r for Mann-Whitney U test: < 0.3 = small, 0.3–0.5 = medium, > 0.5 = large (Karadimitriou et al., 2018).

^***^p < .001

**Table 3:** Spearman’s correlations between age, autistic traits, alexithymia, intolerance of uncertainty, anxiety and the four domains of quality of life in the total sample (*N* = 167)

	1	2	3	4	5	6	7	8	9
1. Age									
2. Autistic Traits	0.25^**^	(0.95)							
3. Alexithymia	0.13	0.72^***^	(0.91)						
4. Intolerance of Uncertainty	0.14	0.78^***^	0.64^***^	(0.95)					
5. Anxiety	0.07	0.55^***^	0.44^***^	0.68^***^	(0.89)				
6. Physical Health QoL	-0.17^*^	-0.55^***^	-0.43^***^	-0.55^***^	-0.66^***^	(0.85)			
7. Psychological Health QoL	-0.04	-0.51^***^	-0.44^***^	-0.54^***^	-0.70^***^	0.71^***^	(0.86)		
8. Social Relations QoL	-0.16^*^	-0.33^***^	-0.35^***^	-0.40^***^	-0.50^***^	0.42^***^	0.63^***^	(0.74)	
9. Environment QoL	-0.04	-0.55^***^	-0.42^***^	-0.56^***^	-0.66^***^	0.71^***^	0.69^***^	0.50^***^	(0.87)

*Note* Numbers in parentheses are Cronbach’s alpha. Autistic Traits were assessed by the 14-item Ritvo Autism & Asperger Diagnostic Scale (RAADS-14).

^*^p < .05; ^**^p < .01; ^***^p < .001

### Mediation Models

**Fig. 2:**
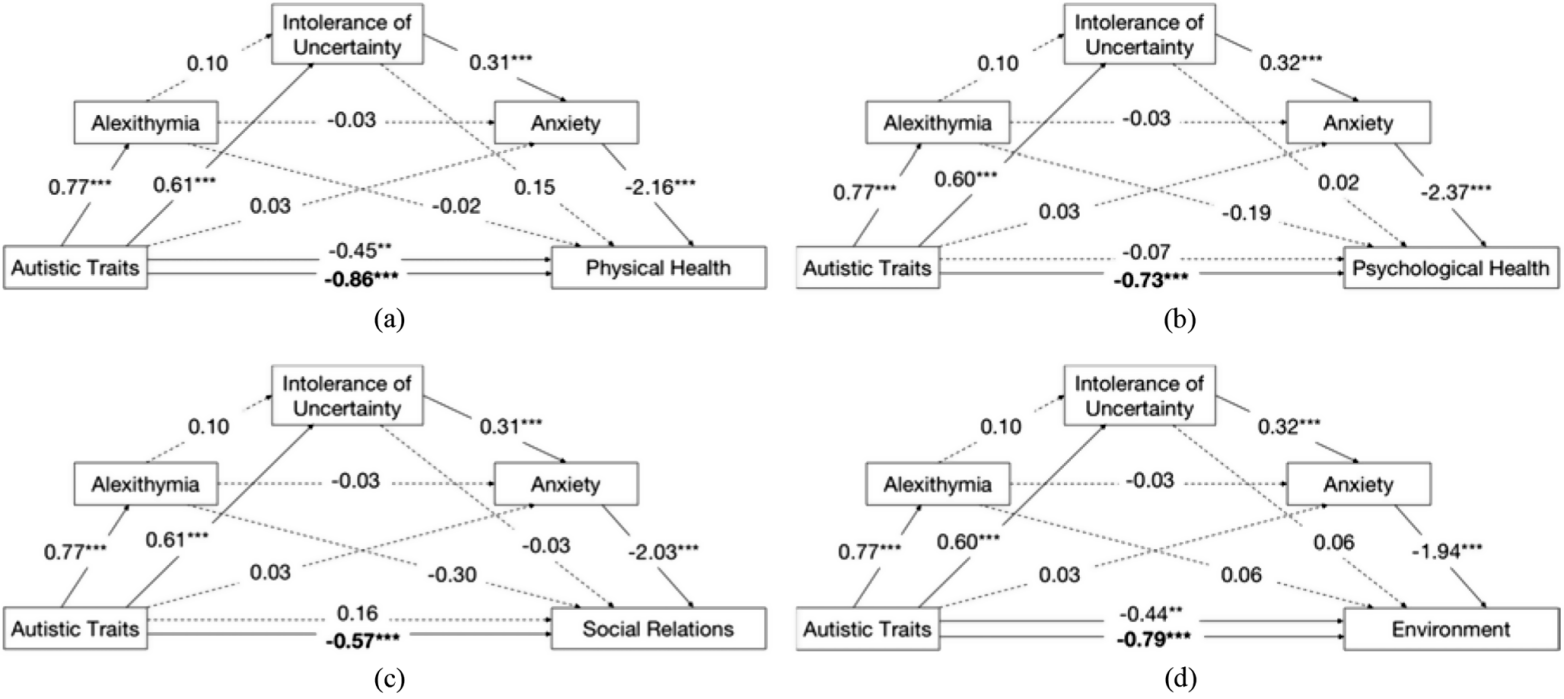
Mediation models from autistic traits to four domains of quality of life via alexithymia, intolerance of uncertainty and anxiety. *Note* Mediation models from autistic traits via alexithymia, intolerance of uncertainty and anxiety to: (**a**) physical health, (**b**) psychological health (**c**) social relations, and (**d**) environment domain of WHOQOL-BREF. Age was controlled for in the mediation models for physical health and social relations domains. The bolded coefficient is the total effect, i.e., c path. Autistic Traits were assessed by the 14-item Ritvo Autism & Asperger Diagnostic Scale (RAADS-14). ^**^p < .01; ^***^p < .001

Figure 2 shows the mediation models for the four domains of QoL. Partially supporting the third hypothesis, IU and anxiety - but not alexithymia - mediated the relationship bewteen autisic traits and QoL in all four domains. Results of the direct effects, indirect effects and total effect for the four serial mediation models with three mediators (Fig. 2 (a) to (d)) are presented in Supplementary Materials 1 to 4.

The pathways from autistic traits to physical health and environment QoL domains were partially mediated by IU and anxiety. For the physical health domain, the total effect of the mediation model was significant (*b* = -0.86, $\beta$ = -0.56, *SE* = 0.10, *p* < .001) when controlling for age (see Table 4; Fig. 2 (a)). Autistic traits showed a significant direct effect (*b* = -0.45, $\beta$ = -0.29, *SE* = 0.16, *p* = .005), and indirect effect via IU and anxiety (*b* = -0.42, $\beta$ = -0.27, *SE* = 0.10), but not via alexithymia, on physical health. The effect size of the indirect effect was 0.49 on physical health, which meant the IU-anxiety path could explain 49% mediation effect of the relationship between autistic traits and the physical health domain of QoL. For the environment domain, the total effect of the mediation model was significant (*b* = -0.79, $\beta$ = -0.54, *SE* = 0.10, *p* < .001) (see Table 4; Fig. 2 (d)). Autistic traits showed a significant direct effect (*b* = -0.44, $\beta$ = -0.30, *SE* = 0.15, *p* = .004), and indirect effect via IU and anxiety (*b* = -0.37, $\beta$ = -0.25, *SE* = 0.09), but not via alexithymia, on the environment domain of QoL. The effect size of the indirect effect was 0.47 on environment QoL, which meant the IU-anxiety path could explain 47% mediation effect of the relationship between autistic traits and environment domain of QoL.

**Table 4:** Total and Indirect effects of alexithymia, intolerance of uncertainty and anxiety on the relationship between autistic traits and quality of life domain whilst controlling for age (*N* = 167)

Outcome	Model pathways	R^2^	F	b	$\beta$	SE	t	95%CI
Physical Health QoL	Total effect							
	Autistic Traits – Physical Health	0.31	37.60^***^	-0.86	-0.56	0.10	-8.47^***^	[-1.07, -0.66]
	Indirect effect							
	Autistic Traits – Intolerance of Uncertainty – Anxiety – Physical Health			-0.42	-0.27	0.10		**[-0.63, -0.25]**
	Autistic Traits – Alexithymia – Intolerance of Uncertainty – Anxiety – Physical Health			-0.05	-0.03	0.03		[-0.12, 0.01]
Psychological Health QoL	Total effect							
	Autistic Traits – Psychological Health	0.24	51.00^***^	-0.73	-0.49	0.10	-7.14^***^	[-0.93, -0.53]
	Indirect effect							
	Autistic Traits – Intolerance of Uncertainty – Anxiety – Psychological Health			-0.45	-0.30	0.10		**[-0.66, -0.28]**
	Autistic Traits – Alexithymia – Intolerance of Uncertainty – Anxiety – Psychological Health			-0.06	-0.04	0.04		[-0.14, 0.01]
Social Relations QoL	Total effect							
	Autistic Traits – Social Relations	0.12	11.09^***^	-0.57	-0.33	0.13	-4.39^***^	[-0.82, -0.31]
	Indirect effect							
	Autistic Traits – Intolerance of Uncertainty – Anxiety – Social Relations			-0.39	-0.23	0.12		**[-0.65, -0.19]**
	Autistic Traits – Alexithymia – Intolerance of Uncertainty – Anxiety – Social Relations			-0.05	-0.03	0.03		[-0.12, 0.01]
Environment QoL	Total effect							
	Autistic Traits – Environment	0.29	68.92^***^	-0.79	-0.54	0.10	-8.30^***^	[-0.98, -0.60]
	Indirect effect							
	Autistic Traits – Intolerance of Uncertainty – Anxiety – Environment			-0.37	-0.25	0.09		**[-0.56, -0.21]**
	Autistic Traits – Alexithymia – Intolerance of Uncertainty – Anxiety – Environment			-0.05	-0.03	0.03		[-0.12, 0.01]

*Note* Significant indirect effect was noted in bold (95% confidence interval does not cross zero).

Autistic Traits were assessed by the 14-item Ritvo Autism & Asperger Diagnostic Scale (RAADS-14).

^*^p < .05; ^**^p < .01; ^***^p < .001

The associations from autistic traits to psychological health and social relations domains of QoL were fully mediated by IU and anxiety. For the psychological health domain, the total effect of the mediation model was significant (*b* = -0.73, $\beta$ = -0.49, *SE* = 0.10, *p* < .001) (see Table 4; Fig. 2 (b). Autistic traits only had a significant effect on the psychological health domain through the indirect route via IU and anxiety (*b* = -0.45, $\beta$ = -0.30, *SE* = 0.10), but not via alexithymia. The effect size of this indirect effect was 0.62, which meant the IU-anxiety path could explain 62% mediation effect of the relationship between autistic traits and psychological health domain of QoL. For the social relations domain, the total effect of the mediation model was significant (*b* = -0.57, $\beta$ = -0.33, *SE* = 0.13, *p* < .001) when controlling for age (see Table 4; Fig. 2 (c). Autistic traits only had a significant effect on the social relations domain through the indirect route via IU and anxiety (*b* = -0.39, $\beta$ = -0.23, *SE* = 0.12), but not via alexithymia. The effect size of this indirect effect was 0.68, which meant the IU-anxiety path could explain 68% mediation effect of the relationship between autistic traits and social relations domain of QoL. To note, the application of the effect size of the indirect effect (*P*_*M*_) has restrictions on the direction and the values. That is, the coefficient for the total and indirect effect should be in the same direction; the total effect should be big enough to be the denominator divided by the indirect effect. In the present study, all the coefficients were negative, and all the values of the total effect are large enough to reach the requirement of the (*P*_*M*_) effect size (Wen & Fan, 2015). Moreover, the sample size of 167 had 100% power to detect a statistically significant effect (at alpha = 0.05) from autistic traits to QoL in the four domains through the serial IU-anxiety path. Finally, all the models were rerun with the GAFS-8 as the measure for alexithymia (see Supplementary Materials 5–9), and with no change to the results compared to the above findings using the TAS-20 as the alexithymia measure.

## Discussion

In order to investigate whether autistic traits themselves or the associated and co-occurring mental health conditions/thinking styles, play the pivotal role in poor QoL in autistic people, the present study tested a putative mediation model (see Fig. 1). Specifically, we explored whether autistic traits had an indirect effect on QoL through alexithymia, IU and/or anxiety. Replicating previous findings (Hwang et al., 2020; Jolliffe et al., 2022; Larkin et al., 2022), we found support for our first hypothesis that our autistic participants would have higher alexithymia, IU and anxiety than the non-autistic participants. Also supporting our first hypothesis, self-rated QoL across all four domains (i.e., physical health, psychological health, social relations, environment) was lower in the autistic than non-autistic adults, which is consistent with some previous studies (Lawson et al., 2020; Oakley et al., 2021; but for contrasting findings see Ayres et al., 2018). Supporting our second hypothesis, higher autistic traits in the entire sample were associated with, higher alexithymia, higher IU and anxiety, but lower QoL across all four domains, which is consistent with previous findings (Barros et al., 2022; Capp et al., 2022; Jenkinson et al., 2020; Mason & Happé, 2022; Sonido et al., 2022; South et al., 2021; Yarar et al., 2022). In part supporting our final hypothesis, the poor physical health and environment domains of QoL were partially accounted for by autistic traits directly and partially through IU and anxiety (but not alexithymia); and IU and anxiety (but not alexithymia) fully explained the relationship between high autistic traits and poor psychological health and social relations domains of QoL.

How should the different degrees of mediation found for different QoL domains be interpreted? One possible explanation might be that the self-reported symptoms of IU and anxiety emphasise the importance of inner perceptions and feelings, as do items in the psychological health and social relations QoL domains (e.g., self-evaluation, negative emotions). By contrast, the items in the domains of physical health and environment ask for views about external circumstances (e.g., medical treatment, leisure activities). Additionally, item 26 in the psychological domain asks about the frequency of feeling anxiety, which increases the direct link between anxiety and the psychological health domain of QoL. A previous study also supported the specific contribution of anxiety to social QoL; anxiety has been found to moderate the association between the severity of autistic symptoms and QoL in the social relations domain (Smith et al., 2019). Therefore, IU and anxiety more strongly mediates the association between autistic traits and QoL in the psychological health and social relations domains than in the physical health and environment domains.

Alexithymia was not a significant mediator between autistic traits and QoL, counter to our hypothesised model. Inconsistent with previous studies (Barros et al., 2022; Maisel et la., 2016; Ozsivadjian et al., 2021), the significant association between alexithymia and anxiety was not maintained in our mediation model, and the association between alexithymia and IU was no longer significant when entered into the present mediation model. These inconsistent findings might be explained by overlapping features of autism and alexithymia. Ozsivadjian et al. (2021) found that the significant pathway from autistic symptoms to anxiety via IU was later covered by the significant pathway from alexithymia to anxiety via IU when moving alexithymia from the position of mediator to predictor. Consistent with our current study, the significant correlation between alexithymia with anxiety and IU disappeared when autistic traits were entered as a predictor. Moreover, a previous hierarchical regression model also suggested that difficulties in executive functioning (characteristic of autism) but not in emotional processing (more related to alexithymia) accounted for variance in lower subjective QoL in autistic adults (Dijkhuis et al., 2017). In addition, a recent study in the general population found that alexithymia levels did not predict QoL on any of the four WHOQOL domains (Aslan & Batmaz, 2022).

### Strengths and Limitations

There were some strengths for this study. This is the first study using a serial mediation model to integrate autistic traits, co-occurring alexithymia, IU and anxiety, and QoL together to explore their association within a sample of autistic and non-autistic adults. Also, the current findings tested a putative mechanism underlying QoL in autism, and highlight the importance of co-occurring thinking styles and mental health conditions for autistic individuals’ QoL, with possible implications for interventions. Finally, most of the comparison and mediation analyses showed a medium or large effect size, and our study appears to have had sufficient statistical power.

However, there were some limitations. First, perhaps especially for online studies (Rødgaard et al., 2022), our participants may not be fully representative of the wider autistic and comparison populations. As in most volunteer samples, we had a majority female sample in both the autistic and non-autistic groups. Given the increasing attention on autistic women and girls (Hull et al., 2020; Lockwood Estrin et al., 2021; Milner et al., 2019), the present study may still be valuable, but awaits replication in a more sex-balanced sample. Another limitation might be the use of previously collected data from a study on a specific topic, moving home. As moving home is a common experience, we do not foresee major artifacts in the data from recruitment to that topic, but replication (with a different recruitment topic) is needed. Also, we relied on self-reported autism diagnosis without confirmation from clinicians, and included in our autism group a small number of participants who self-identified as autistic. For this reason, and also because of possible under-diagnosis in older adults, we used two autism trait measures to characterize the subgroups (self-identified autistic individuals reported high autistic traits, and the diagnosed autism group and self-identified group reported significantly greater proportions that passed trait measure cut-off scores and higher trait measure total score relative to the non-autistic group; see Supplementary Material 10). Therefore, the present study captured a reasonable sample to represent the range of autistic traits across non-autistic, subclinical high-trait, and autistic adults.

We note current debate in the literature about how QoL should be conceptualized and measured in autistic groups, and future studies might take complementary approaches; here we used a measure whose psychometric properties have been well established for autistic samples, and which showed good internal consistency in our data. Finally, the current study is a cross-sectional study and as such the causal associations suggested are only putative. Although we based our models on previous studies (e.g., Ozsivadjian et al., 2021; Sonido et al., 2021; South et al., 2021) and found largely consistent associations, the statistically significant predictive relationships reported here remain to be tested through longitudinal and/or intervention designs within clinical and non-clinical groups.

### Implications

If replicated, the current results have some potentially important implications for future practice and research. First, IU may be a useful target not only for reducing anxiety in autism (Rodgers et al., 2022) but also for improving QoL. However, improving QoL may need supports tailored to each domain, which may differ in the balance of internal (e.g., thinking style) and external (e.g., environmental) strategies and supports.

In addition, relative level of awareness of own emotions (e.g., alexithymia) may not be as important for QoL as other factors. It may be that this is a distal variable that only weakly impairs perceptions of QoL. Mason and Happé (2022) found that alexithymia did predict poorer QoL, but when adding in depression/anxiety this effect disappeared. Marchesi et al.’s (2000) study, using factor analysis in a sample of 113 patients with depressive and anxiety disorders and 113 control subjects, supported the possibility that the “difficulty identifying feelings” aspect of alexithymia (tested by the TAS-20) overlaps to some extent with anxiety (tested by the 14-items Hospital Anxiety and Depression Scale). Therefore, examination of “pure” alexithymia (i.e., alexithymia in the absence of depression/ anxiety) might be helpful for future researchers to explore further the possible mechanisms between emotional awareness and QoL.

Furthermore, the present mediation models suggests that lower QoL is not the result of autism/autistic traits per se. In addition to co-occurring mental health conditions and thinking styles, it is important to recognise the many factors in the world that effect autistic people and might lead to poorer QoL. For example, factors such as employment status, relationship status, social experience of autism-related stigma, and lack of external support regarding health and financial conditions, have been found to be significant predictors of QoL in autistic adults across cultures (Caron et al., 2022; Mason et al., 2018). All these factors are not solely “belonging to” the autistic person, they are “belonging to” the wider society, and wider social policies and attitudinal change are needed to address them.

## Conclusison

Overall, in the current study, autistic adults reported poorer subjective QoL than non-autistic adults in the physical health, psychological health, social relations and environment domains. Across the total sample, significant negative associations between levels of autistic traits and the four domains of QoL were found to be mediated serially through IU and anxiety (but not alexithymia). In the physical health and environment domains, autistic traits appeared to have both direct and indirect effects, via IU and anxiety, on QoL. In the psychological health and social relations domains, the effects of autistic traits on QoL were entirely mediated by IU and anxiety. These findings suggest that IU and resultant anxiety may be good intervention targets in order to improve autistic people’s QoL. Policies, interventions and treatments, and social service and support for autistic people should emphasize the key role of co-occurring mental health conditions; poor QoL does not have to follow from autism or high autistic traits.
